# Effects of hypoxia on Achilles tendon repair using adipose tissue-derived mesenchymal stem cells seeded small intestinal submucosa

**DOI:** 10.1186/s13018-021-02713-x

**Published:** 2021-09-24

**Authors:** Xing Guo, Hui Lv, ZhongWei Fan, Ke Duan, Jie Liang, LongFei Zou, Hao Xue, DengHua Huang, YuanHui Wang, MeiYun Tan

**Affiliations:** 1 Department of Orthopaedics, Affiliated Hospital of Southwest Medical University, Luzhou, 646000 Sichuan China; 2 Sichuan Provincial Lab of Orthopaedic Engineering, Luzhou, 646000 Sichuan China; 3 Department of Orthopaedic Surgery, The First People’s Hospital of Neijiang, Neijiang, 641100 Sichuan China

**Keywords:** Adipose-derived mesenchymal stem cells, Small intestinal submucosa, Hypoxic, Achilles tendon, Tissue engineering

## Abstract

**Background:**

The study was performed to evaluate the feasibility of utilizing small intestinal submucosa (SIS) scaffolds seeded with adipose-derived mesenchymal stem cells (ADMSCs) for engineered tendon repairing rat Achilles tendon defects and to compare the effects of preconditioning treatments (hypoxic vs. normoxic) on the tendon healing.

**Methods:**

Fifty SD rats were randomized into five groups. Group A received sham operation (blank control). In other groups, the Achilles tendon was resected and filled with the original tendon (Group B, autograft), cell-free SIS (Group C), or SIS seeded with ADMSCs preconditioned under normoxic conditions (Group D) or hypoxic conditions (Group E). Samples were collected 4 weeks after operation and analyzed by histology, immunohistochemistry, and tensile testing.

**Results:**

Histologically, compared with Groups C and D, Group E showed a significant improvement in extracellular matrix production and a higher compactness of collagen fibers. Group E also exhibited a significantly higher peak tensile load than Groups D and C. Additionally, Group D had a significantly higher peak load than Group C. Immunohistochemically, Group E exhibited a significantly higher percentage of MKX + cells than Group D. The proportion of ADMSCs simultaneously positive for both MKX and CM-Dil observed from Group E was also greater than that in Group D.

**Conclusions:**

In this animal model, the engineered tendon grafts created by seeding ADMSCs on SIS were superior to cell-free SIS. The hypoxic precondition further improved the expression of tendon-related genes in the seeded cells and increased the rupture load after grafting in the Achilles tendon defects.

## Introduction

Achilles tendon rupture is a common injury of the musculoskeletal system. In the UK, it is estimated to affect 0.113% of the population annually [1]. Current treatments include drug administration, physiotherapy, and autografting, but the outcome is frequently unsatisfactory as the tendon is poorly vascularized and cellularized. Autografting is relatively effective in restoring the function of damaged tendons, but its use is limited by the availability of autografts and donor-site damage [2, 3]. Tissue engineering is emerging as a new option of managing tendon injuries. In this technique, cells are seeded on a scaffold and cultured in vitro to induce their proliferation and differentiation to a desired phenotype, creating an analog of autograft for implantation [4]. The scaffold material, cells, and culture conditions are all critical for the success of this technique.

Many materials have been studied for tendon tissue engineering, such as polypropylene mesh, human amniotic membrane (HAM), and small intestinal submucosa (SIS). Polypropylene mesh is commercially available and has generally satisfactory mechanical properties, but it is non-degradable in vivo. HAM supports cell adhesion, migration, and proliferation; unfortunately, its biomechanical properties are unsatisfactory, especially in the early stage of healing [5]. SIS is an extracellular matrix (ECM) containing abundant collagen fibers, proteoglycans, and growth factors [6] that play important roles in tissue repair [7–10]. In vitro, SIS was reported to promote the adhesion, proliferation, and differentiation of stem cells [11]. In vivo, SIS-based scaffolds have been studied for repairing defects of tissues such as the vocal cords, dura mater, and tendons [7–10].

Mesenchymal stem cells (MSCs) are multipotent cells able to self-renew while remaining undifferentiated [12]. Studies have demonstrated that, under appropriate conditions, MSCs can differentiate into different phenotypes such as osteoblasts, chondrocytes, and fibroblasts [13, 14]. Bone marrow-derived mesenchymal stem cells (BMSCs) have been commonly used as seed cells for the engineering of various tissues such as the bone, myocardium, and tendon [15–17]. Nevertheless, BMSCs are associated with technical disadvantages such as invasive procedures of isolation and low BMSC concentration in the bone marrow [18]. Recently, MSCs derived from adipose tissue (ADMSCs) have gained increasing attention because of their less invasive harvesting, relatively abundance, and higher proliferative capacity [19]. Furthermore, ADMSCs are particularly suitable for tissue engineering when a large number of seed cells are required. In an injury model of horse flexor tendonitis, after injection of ADMSCs, the tissue structure of collagen fibers was improved and the properties of tendons were enhanced [20]. In a rabbit model, defects of Achilles tendons were repaired with platelet-rich plasma (PRP) or a mixture of PRP and ADMSCs. At week 4, the tendons repaired with the PRP-ADMSCs mixture were significantly stronger than those receiving PRP alone [21]. These results suggest ADMSCs to be promising cells for the repair of soft tissues. Currently, however, no study has investigated the use of ADMSCs combined with SIS for the tissue engineering of tendons.

In addition to scaffolds and seed cells, the environment in which the cells proliferate and differentiate is also crucial for the success of tissue engineering. For example, hypoxic conditions have been found to promote the secretion of factors (e.g., vascular endothelial growth factor, hypoxia-inducible factors) by MSCs and, thus, considered a potential strategy to improve the survival of transplanted MSCs [22]. Huang et al. [23] cultured BMSCs under hypoxic or normoxic conditions, and injected the cells into experimental defects created in rat Achilles tendons. Four weeks after injection, the defects repaired with BMSCs cultured under hypoxic conditions showed a significantly higher ultimate failure load than those repaired with the cells cultured under normoxic conditions. Other animal studies found that, hypoxic preconditioning of ADMSCs promoted wound healing, cartilage repair, and angiogenesis [24–26]. However, no study has investigated the effects of hypoxic conditioning on tendon tissue engineering with ADMSCs.

The present study investigated the repair of rat Achilles tendon defects with SIS scaffolds seeded with ADMSCs, and compared the effects of preconditioning treatments (hypoxic vs. normoxic) on the tendon healing. Our goal was to generate fundamental information for improving the outcomes of tendon tissue engineering.

## Materials and methods

### Animals

Sixty male Sprague–Dawley rats (8–10 weeks, 250–300 g) were purchased from Experimental Animal Center of Southwest Medical University. The study protocol was approved by Committee of Research Ethics of Southwest Medical University, and all procedures followed Experimental Animal Welfare Guidelines of the University.

### Cell isolation

The animal was anesthetized by intraperitoneal injection of 2% (w/w) pentobarbital (30 mg/kg). Inguinal adipose tissue was surgically collected bilaterally under aseptic conditions. The tissue was washed three times with PBS, fragmented, and digested with 0.1% type-I collagenase (Sigma, St Louis, MO, USA) under continuous shaking (37 °C, 60 min). Digestion was terminated by adding equal volume of complete culture medium (CCM; a-MEM supplemented with 16.6% fetal bovine serum, 100 U/mL penicillin, 0.1 g/mL streptomycin, and 2 mM L-glutamine). The resultant mixture was filtered with a mesh (pore size: 75 µm; Sigma), and the filtrate was diluted with CCM and centrifuged (1500 r/min, 5 min). The supernatant was aspirated off, and the cells were resuspended in PBS and centrifuged again (1500 r/min, 5 min). After removing the supernatant again, the cells were resuspended in CCM, seeded in Petri dishes (1 × 10^5^ cell/cm^2^), and incubated (37 °C, 5% CO_2_, 20% O_2_, relative humidity 95%) with a renewal of CCM every 3 days.

### Flow cytometry

When the cells at passage 3 reached 80–90% confluency, the medium was replaced with 2 ml of 0.25% trypsin (Gibco). After digestion for 30 min, the cells were transferred to a 15-ml Eppendorf tube and treated by two cycles of: centrifugation (5 min, 1000 r/min), supernatant removal, and resuspension of the pellet in 5 ml of PBS. The antibodies to CD29-APC, CD90-PE-Cy7, CD34- PE, CD45-PerCP, CD31-PE and homotype control antibody were added into the suspension. After incubation (room temperature, 0.5 h), the cells were analyzed by flow cytometry (BD FACSAria, BD, San Jose, CA, USA).

### Cell preconditioning

Cells at passage 3 were seeded in Petri dishes (1 × 10^5^ cell/cm^2^) and incubated under either normoxic (20% O_2_, 75% N_2_, 5% CO_2_) or hypoxic conditions (2% O_2_, 93% N_2_, and 5% CO_2_) for up to 7 days.

### Fluorescence labeling

To track the differentiation of ADMSCs in vivo, the cells were labeled with Dilute Vybrant CM-DiI cell-labeling solution (Invitrogen, Carlsbad, CA, USA). Subsequently, the cells were incubated at 37 °C for 30 min, and then at 4 °C for 15 min.

### Preparation of SIS scaffolds

Porcine SIS was prepared following a previous study [27, 28]. Briefly, porcine jejunum was obtained from a local market, cut into strips (length: ~ 10 cm), and rinsed carefully with saline. The *tunica serosa* and *tunica muscularis* were harvested by mechanical scraping, defatted by immersion in a solution of methanol/chloroform (v: v = 1:1) for 12 h, rinsed repeatedly with distilled water, and disinfected with 0.1% peracetic acid for 30 min. All samples were freeze-dried at − 55 °C for 48 h. The obtained SIS was kept in vacuum-sealed bags and sterilized by ethylene oxide fumigation.

### Cell seeding

The SIS strips were cut into rectangular samples (~ 1 × 1.5 cm) and rehydrated by placing in 6-well plates containing PBS for 24 h. The labeled cells were added into the culture medium and seeded on the SIS samples (1.5 × 10^6^ cells/scaffold) according to our previous experience [27, 28]. Then, they were cultured (37 °C, 5% CO_2_, 20%O_2_) for 48 h.

### Animal grouping and operation

**Fig. 1:**
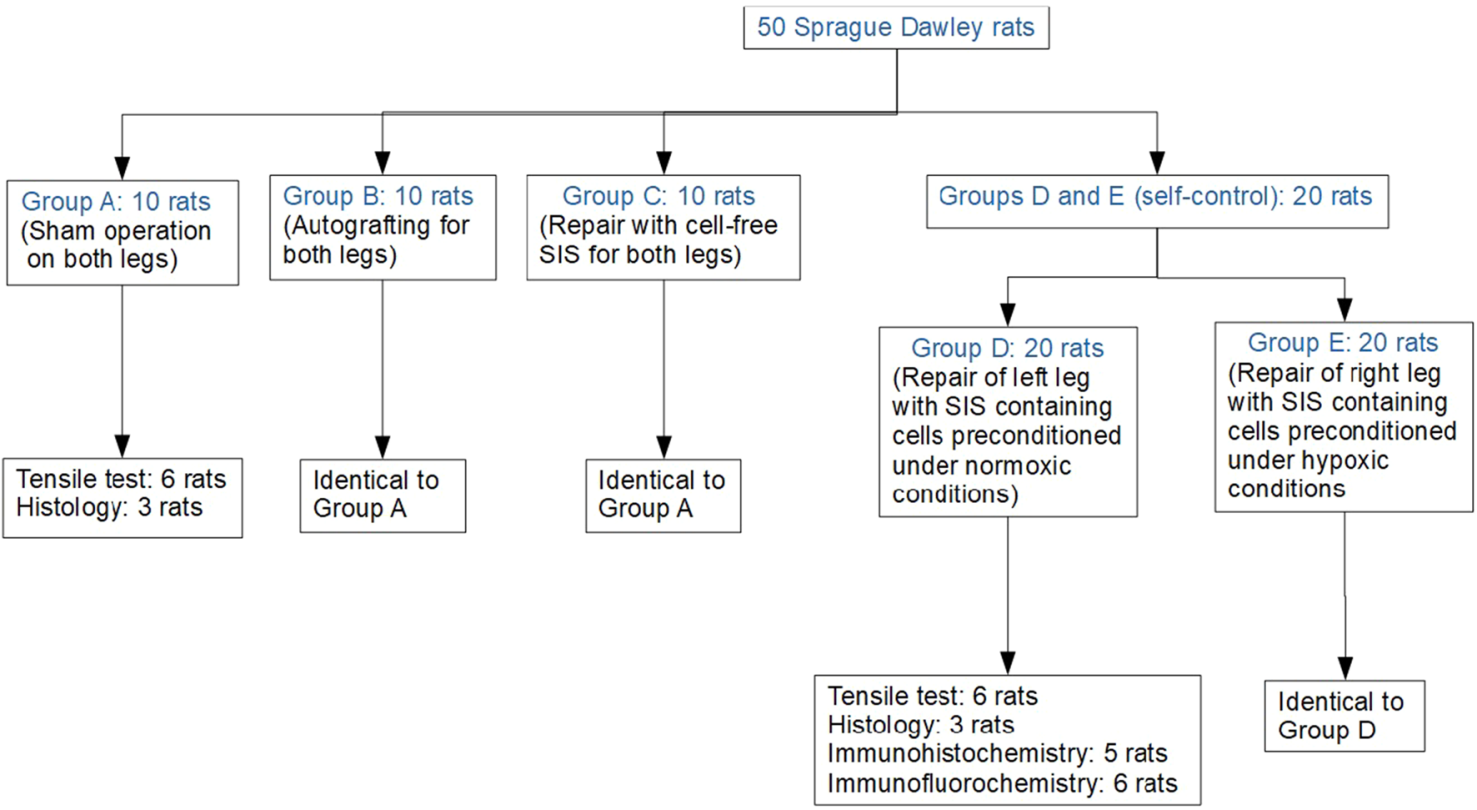
Schematic showing grouping of animals

**Fig. 2:**
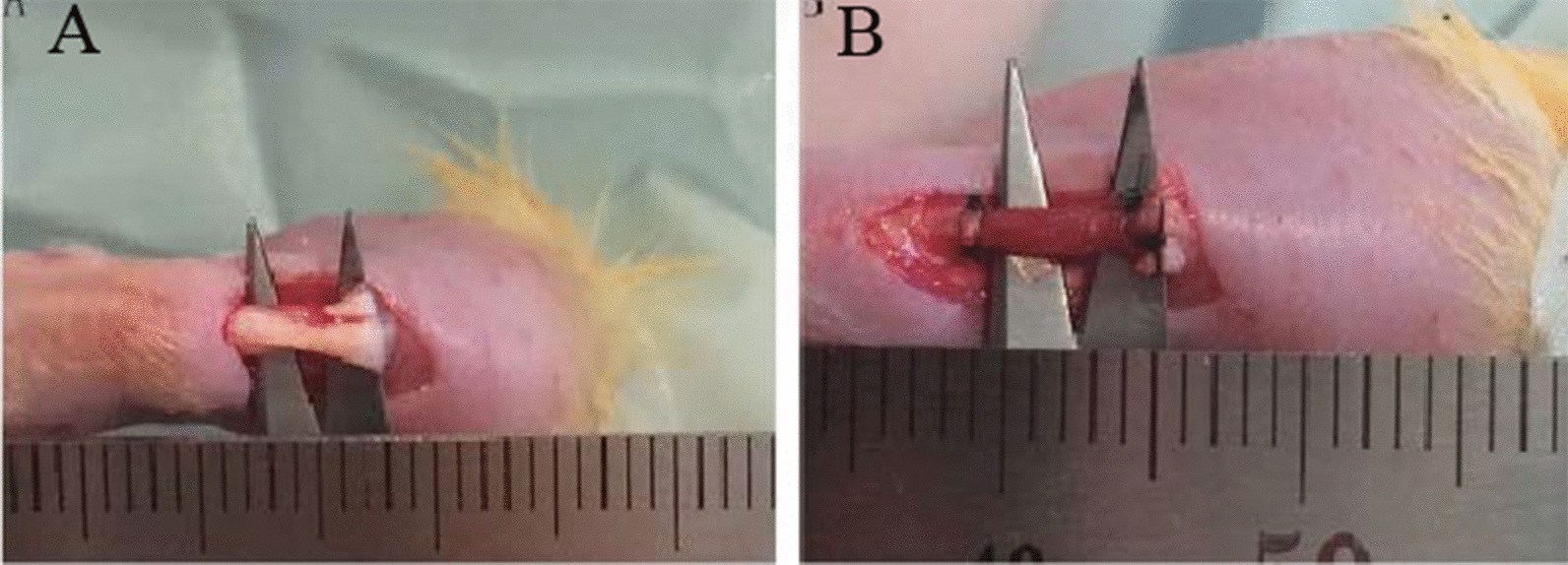
Photographs showing the experimental procedures; **A** surgical exposure of the Achilles tendon to be resected; **B** tissue-engineered graft (in this case an ADMSCs-seeded SIS) sutured to the defect in replacement of the resected tendinous tissue

Fifty SD rats were randomized into five groups (n = 20/group) as shown in Fig. 1. The animal was anesthetized by intraperitoneal injection of sodium pentobarbital (30 mg/kg). A longitudinal incision was made on the posterior aspect of each hind leg above the ankle to expose the Achilles tendon. For Group A (sham operation), the wound was simply closed layer by layer. For the other four groups, a 5-mm segment was resected from the mid-tendinous region (Fig. 2A). For Group B (autograft), the defect was rinsed with 2 ml of penicillin (1: 10,000) and 3 ml of lincomycin (1: 1000). The removed segment was immersed in penicillin (1: 10,000) and lincomycin (1: 1000) solutions each for 15 min, and then sutured back to the original location by a modified Kessler method. For the other three groups, bilateral defects were created similarly; SIS scaffolds were rolled into cylinders (diameter: ~ 2–2.5 mm, length: 5 cm), placed into the defects (Fig. 2B), and sutured identically to Group B. The scaffolds used in Group C were cell-free (Fig. 1), and those in Group D and E were loaded with ADMSCs (1.5 × 10^6^ cells/scaffold) preconditioned under normoxic or hypoxic conditions (Sect. 2.4).

Four weeks after operation, all rats were killed by intravenous injection of pentobarbital (100 mg/kg). All Achilles tendons were harvested for biomechanical and histological evaluations. Samples retrieved from Groups D and E were also investigated by immunohistochemical and immunofluorometric examinations.

### Sample processing and examinations

For histology, harvested samples were immediately fixed in 4% paraformaldehyde, dehydrated in ethanol series, embedded in paraffin, and sectioned sagittally (thickness: ~ 5 µm). The sections were stained with hematoxylin–eosin (HE) and Masson’s Trichrome reagents.

For immunohistochemical examination, paraffin-embedded sections from Groups D and E were de-paraffinized with xylene and rehydrated. They were immersed in 3% H_2_O_2_/methanol (v: v = 1:9) for 15 min to block endogenous peroxidase and rinsed thrice with PBS. Subsequently, the sections were probed with the primary monoclonal antibodies [rabbit antibody to Tenomodulin (Abcam) and rabbit antibody to Mohawk homeobox (Sigma)]. After three rinses with PBS, they were probed again with Dako REAL EnVision detection kits (Dako, Glostrup, Denmark) following manufacturer instructions. Then, diaminobenzidine (Boster Bio, Pleasanton, CA, USA) was added for color development, and the sections were counterstained with hematoxylin.

For immunofluorescent examination, the samples were cryogenically sectioned (thickness: 10 µm). The sections were fixed in cold acetone, rinsed thrice with PBS, and probed sequentially with rabbit antibody to CD31 (Abcam) and rabbit antibody to Mohawk homeobox (Sigma) (each 37 °C for 2 h). After three rinses with PBS, they were probed with fluorescein isothiocyanate (FITC)-labeled goat- anti-rabbit IgG (secondary antibody, 37 °C, 1 h). Finally, they were counterstained with 4',6-diamidino-2-phenylindole (DAPI). Stained sections were observed under a fluorescence microscope, and five random fields were taken and counted for triple-immunostained cells.

For biomechanical test, the samples were harvested from the calcaneus to 1 cm above the proximal suture and rinsed carefully with saline. The collected sample was further dissected to remove irrelevant connective tissues. Thirty grafts (six specimens/group) were randomly selected and tested by tension to rupture (10 mm/min; Instron 5967, load cell: 100 N; Instron, Norwood, MA, USA). Both ends of the sample were secured in a screw grip. The peak failure load was recorded, and the site of sample rupture was observed.

### Statistical analysis

Data from two groups were compared by *t* test (SPPSS 17.0, SPSS, Chicago, IL, USA). Data from > 2 group were analyzed by analysis of variance (ANOVA) and Tukey multiple comparison test. A *p* value < 0.05 was considered statistically significant.

## Results

### ADMSCs culture and identification

**Fig. 3:**
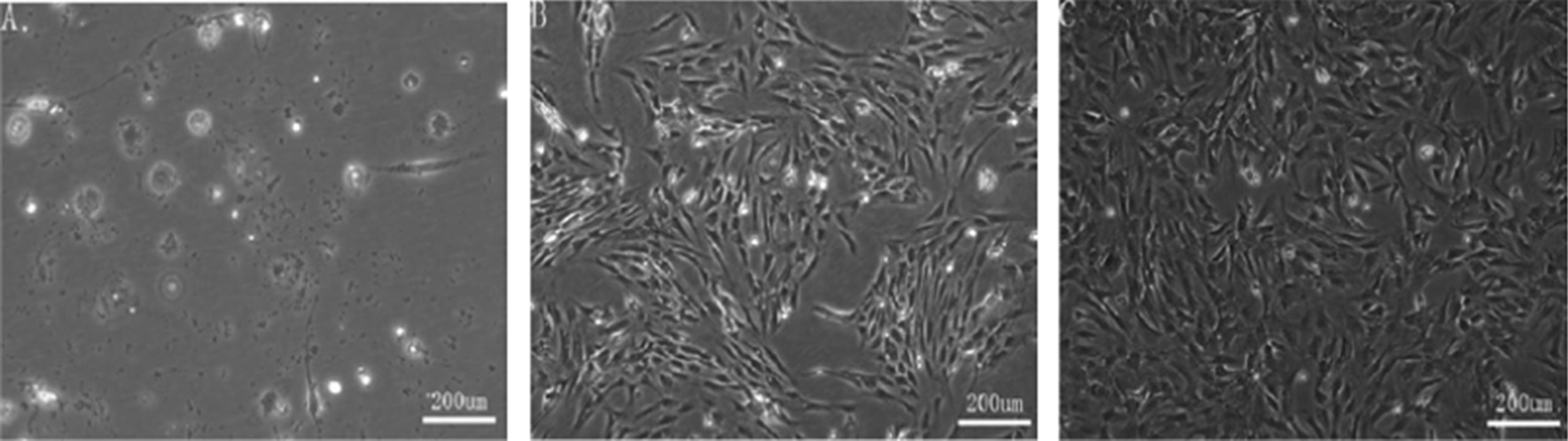
Micrographs of **A**, **B** primary ADMSCs cultured for 24 h and **B** 7 days and **C** cells at the third passage

**Fig. 4:**
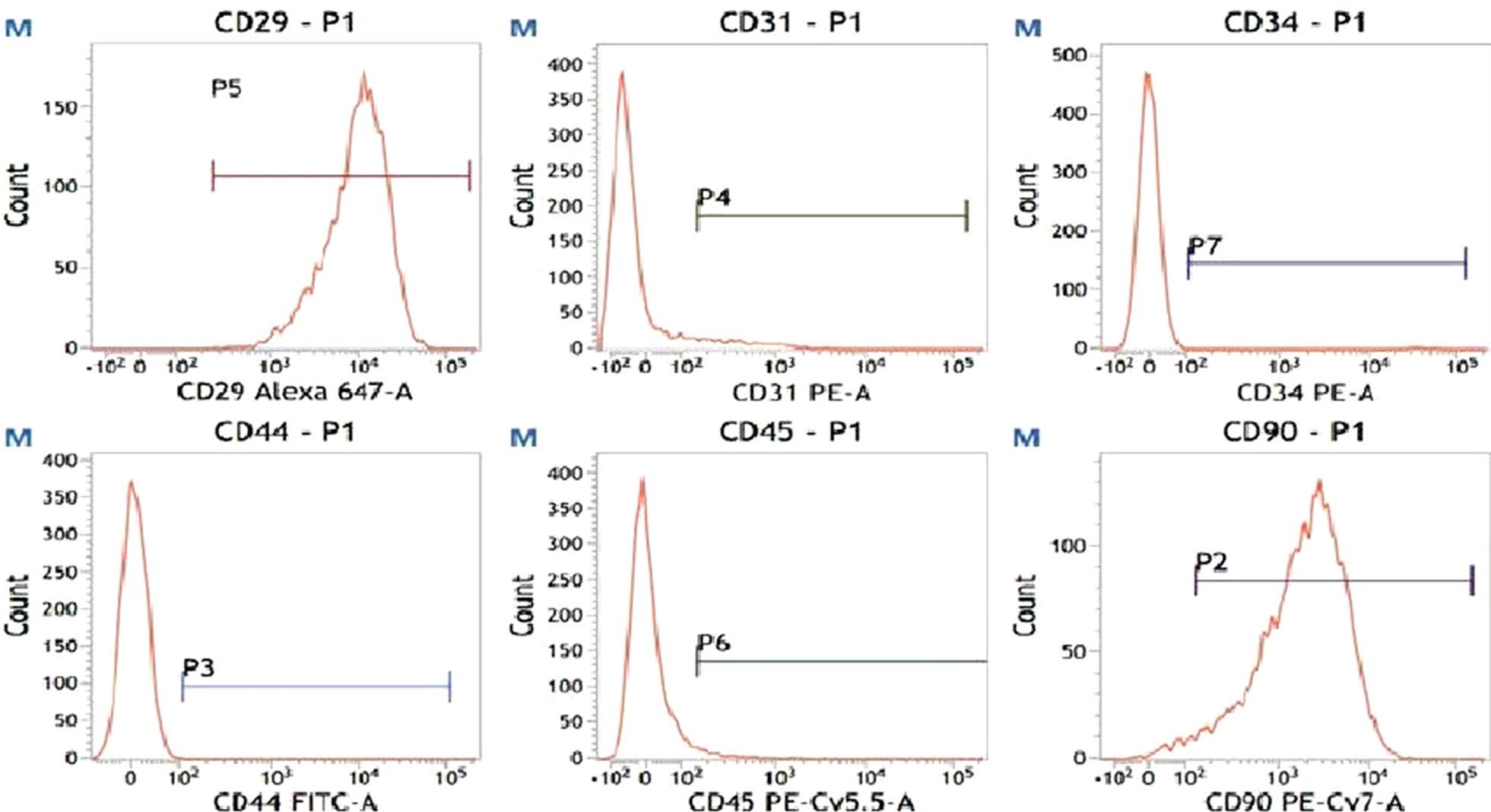
Identification of ADMSCs surface markers

After culture of the primary ADMSCs for 24 h (Fig. 3A), the cells adhered to culture dishes and grew in colonies; most cells appeared round. After culture for 7 days, most cells became spindle-like (Fig. 3B). After three passages, the cells maintained the spindle-like morphology. Flow cytometry found that, > 95% of the cells were CD90^+^ and CD29^+^, whereas < 5% were CD31^−^, CD34^−^, and CD45^−^ (Fig. 4). These characteristics compare favorably with the surface marker profiles of MSCs reported by other studies [29, 30].

### Gross examination

Four weeks after operation, all grafts (Groups C–E) or the native tendon (Group A) showed different degrees of adhesion to the surrounding fascia, with the lightest adhesion observed in Group A and most severe adhesion in Group B. Groups C, D, and E had similar degrees of adhesion. All grafts (Groups C–E) were connected intimately to the adjacent tendinous tissue.

### Histology

#### HE staining

**Fig. 5:**
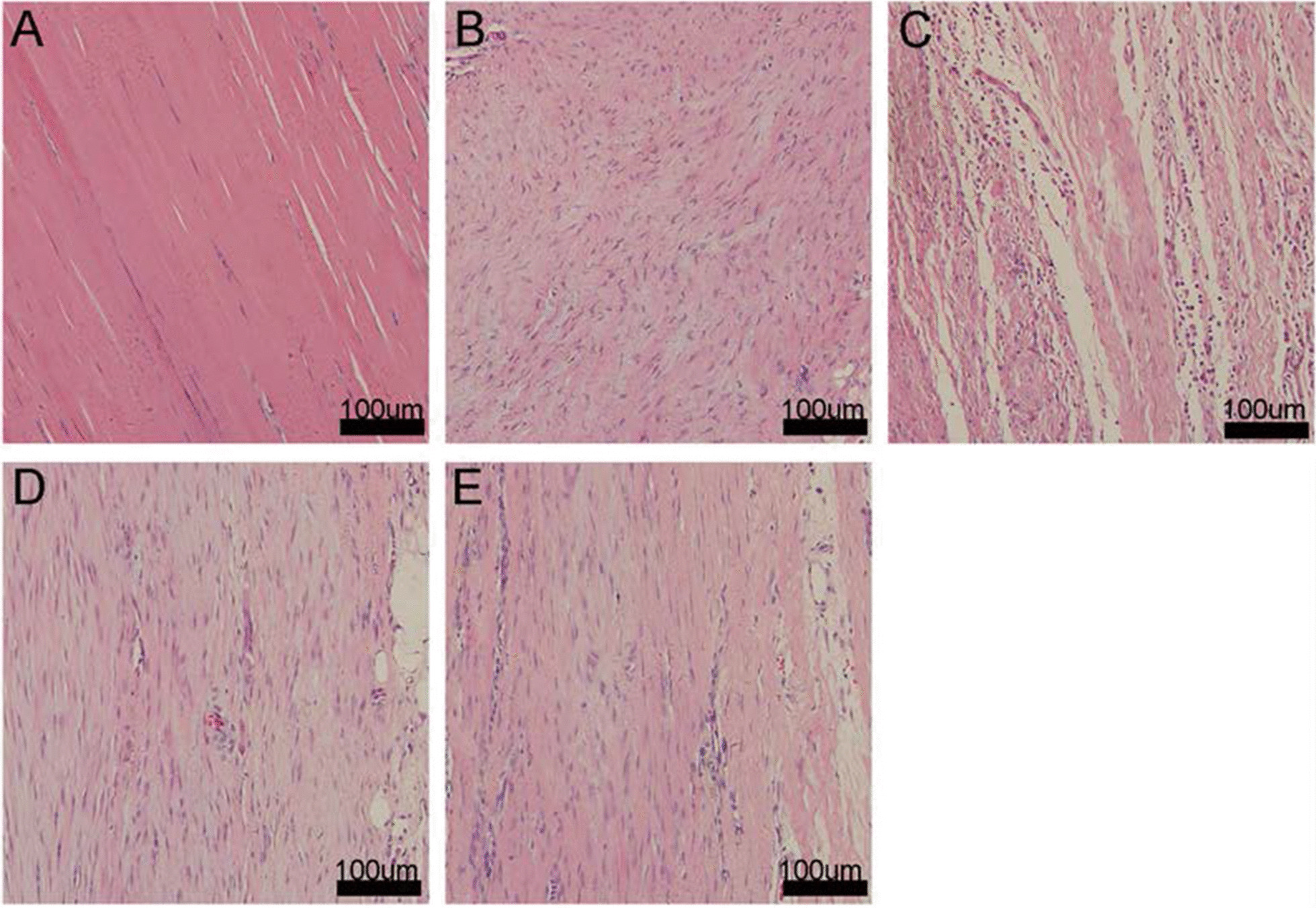
Photomicrographs of sections from Groups A–E (HE staining)

The sections of Group A (Fig. 5A) showed a small number of spindle-shaped tenocytes distributed in a dense matrix of oriented collagen fibers. Group B had abundant spindle-shaped cells distributed in collagen fibers lacking an orientation (Fig. 5B). Group C–E all showed abundant cells aligned along a matrix of oriented collagen fibers (Fig. 5C–E). In Group C, the majority of cells were rounded with a small proportion appearing spindle-shaped, whereas in Groups D and E the cells were predominantly spindle-shaped. Groups D and E showed generally oriented collagen fibers and increased extracellular matrix (ECM) production compared with Group C, with Group E outperforming Group D in ECM content. Moreover, Group D appeared to display a slightly greater cellularity than Group E. The difference between the two groups needs to be further analyzed by immunohistochemistry.

#### Masson’s trichrome staining

**Fig. 6:**
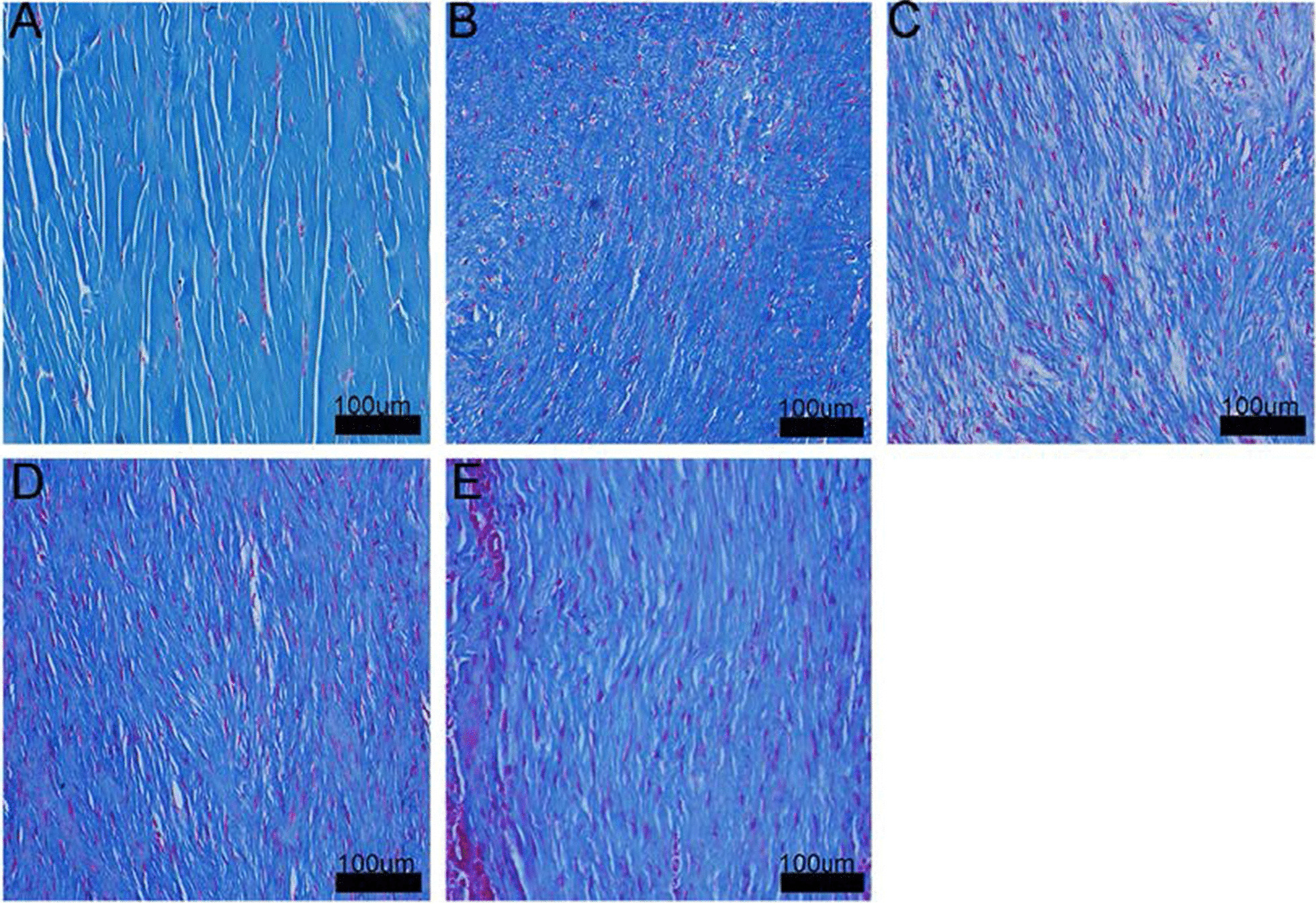
Photomicrographs of sections from Groups A to E (Masson trichrome staining, 4 weeks after operation)

After Masson’s trichrome staining, collagen fibers stained blue; the cytoplasm red; and the nuclei black (Fig. 6). In Group A, a small number of cells were dispersed in compact and well oriented collagen fibers. In Group B, cells were distributed along collagen fibers that were less oriented (vs. Group A). Groups C–E appeared generally similar, with cells distributed along collagen fibers arranged in parallel bundles. Careful observation suggested that, the compactness of collagen fibers appeared to vary slightly, following an order of Group E > Group D > Group C.

### Immunohistochemical staining

Tenomodulin (Tnmd) has been identified as a type-II transmembrane glycoprotein principally expressed in tendons and ligaments [31]. Transcription factor Mohawk homeobox (MKX) is an important regulator of tenogenic differentiation [32, 33]. Recent studies reported that, collagen type I expression and Achilles tendon in mice were defective when the MKX gene was knocked out [32, 34]. Given the importance of Tnmd and MKX in tendon development, we performed immunohistochemical staining of the two proteins to compare the outcome of tendon repair in Groups D and E.

**Fig. 7:**
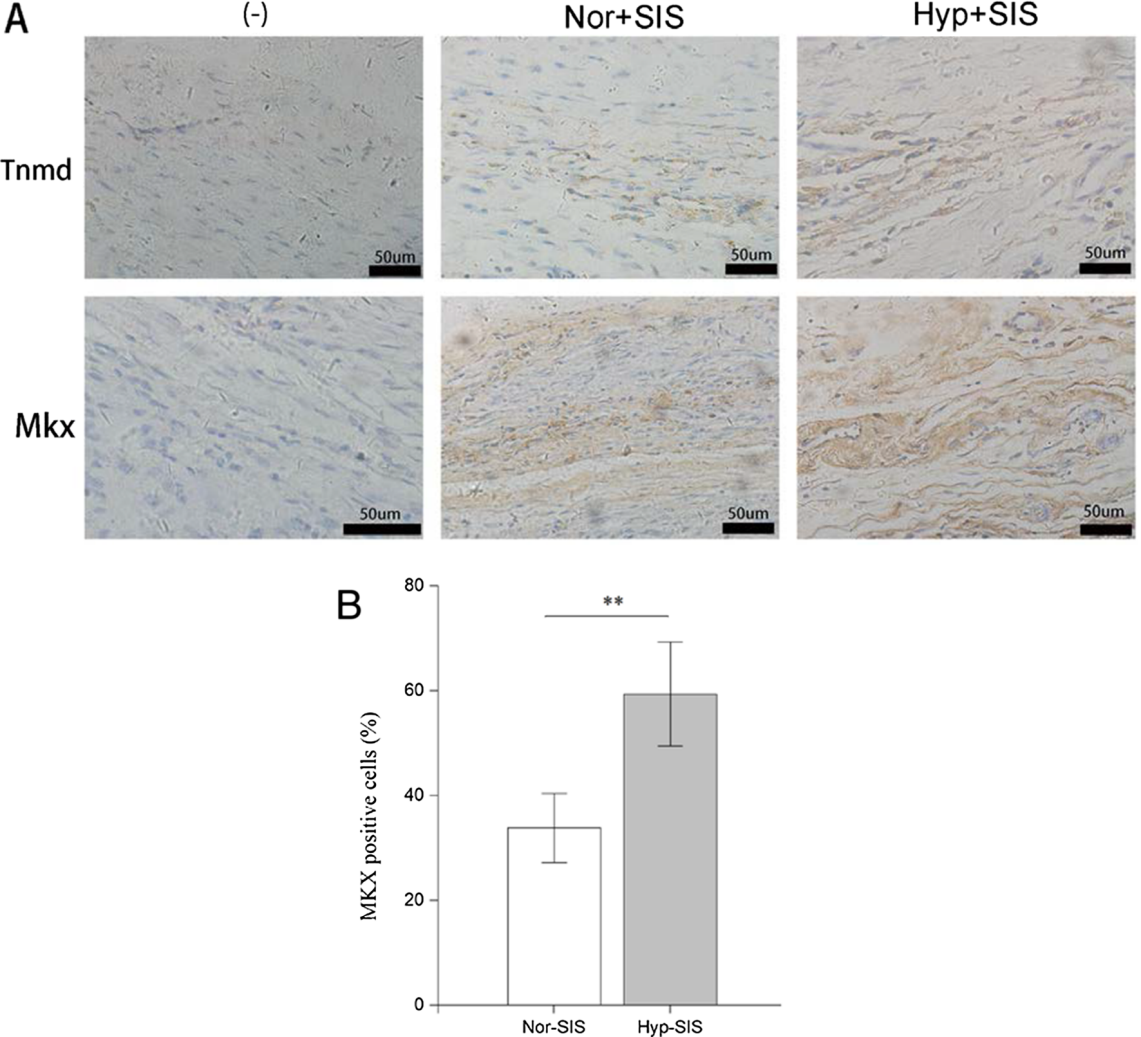
**A** Photomicrographs of sections from Groups D and E immunohistochemically stained for Tnmd and MKX; **B** Comparison of the proportions of MKX + cells measured from photomicrographs in **A** (n = 5). (**p < 0.01)

Four weeks after operation, extensive production of Tnmd and MKX was detected in the regenerated tendon-like tissues in Groups D and E (Fig. 7A), with Group E exhibiting a more intense (vs. Group D) staining of Tnmd and MKX. Image analysis of the sections found that, Group E had a significantly higher percentage of MKX^+^ cells than Group D (*p* = 0.001) (Fig. 7B). This suggests that, compared with Group D, Group E supported an improved generation of tendon-like tissue in this model.

### Immunofluorescent staining

**Fig. 8:**
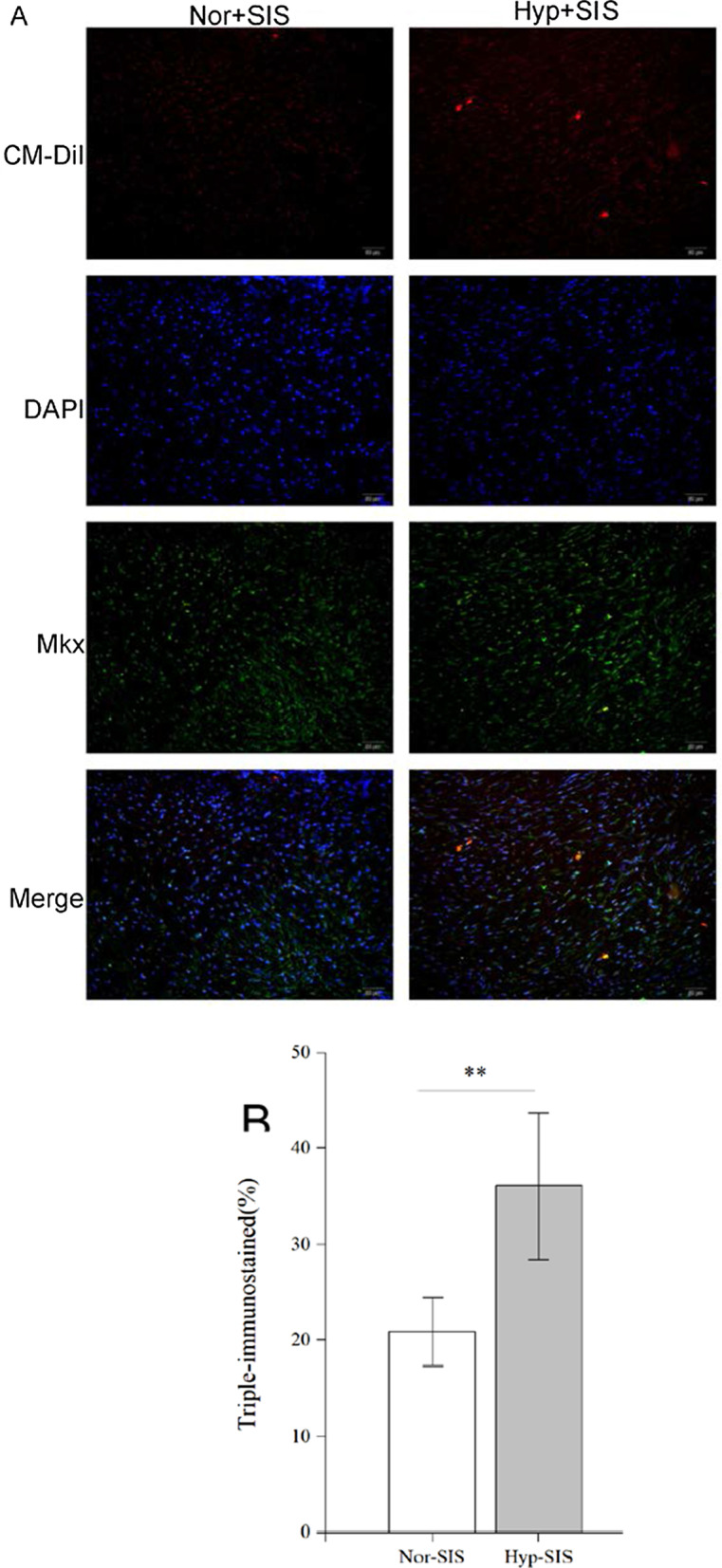
**A** Photomicrographs of sections from Groups D and E immunofluorescently stained for MKX; **B** comparison of the proportions of MKX^+^ cells measured from photomicrographs in **A** (n = 5) (**p < 0.01)

Four weeks after operation, numerous CM-Dil-labeled ADMSCs were seen in sections from Groups D and E, with those in Group E appearing more aligned and narrower. MKX^+^ cells were also detected in the two groups by triple immunofluorescence microscopy (Fig. 8A). Image analysis found that, compared with Group D, Group E had a higher proportion of ADMSCs simultaneously positive for both MKX and CM-Dil (*p* = 0.003) (Fig. 8B). These findings suggest that, ADMSCs seeded in the SIS scaffolds differentiated into MKX^+^ cells in the regenerated tendon-like tissue, and the hypoxic preconditioning enhanced their differentiation.

### Tensile load

**Fig. 9:**
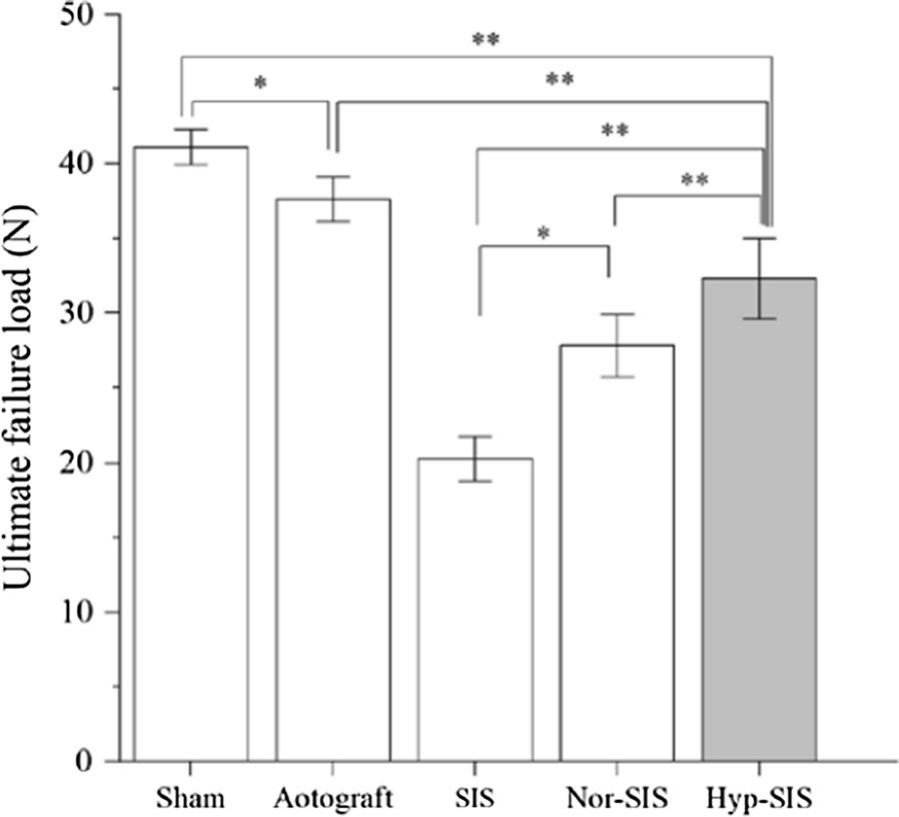
Ultimate tensile failure loads measured at Week 4 (n = 6) (*p < 0.05, ^**^p < 0.01)

Four weeks after operation (Fig. 9), the peak load (i.e., force at rupture) of samples from Group E (32.34 ± 2.71 N) was significantly higher than those from Group D (27.78 ± 2.11 N) (*p* = 0.003) and Group C (20.33 ± 1.47 N) (*p* < 0.001). The ultimate load recorded from Group D was significantly higher than that that of Group C (*p* < 0.001). Additionally, the ultimate load of Group A (41.12 ± 1.23 N) was significantly higher than that of Group B (37.62 ± 1.54 N) (*p* = 0.027). The ultimate load of Group E reached 78% (*p* < 0.001) of that of Group A and 85% (*p* < 0.001) of that of Group B, although both differences remained statistically significant. All samples failed at the middle third. These indicate that, seeding of ADMSCs significantly increased the strength of the regenerated tendon-like tissue (vs. grafting of cell-free SIS scaffolds) at Week 4, and the hypoxic preconditioning further significantly improved the strength.

## Discussion

The current study found that, (1) compared with the grafts of cell-free SIS, grafting of SIS containing seeded ADMSCs yielded tendon-like tissue with improved histological and mechanical properties, and (2) compared with normoxic preconditioning, hypoxic pre-conditioning of ADMSCs increased the ultimate load and histological characteristics of the new tissue.

SIS is a natural, acellular ECM hosting a partially aligned collagen type-I matrix and a variety of growth factors [35]. Previous studies reported positive results from the use of cell-free SIS in managing soft tissue defects in animal models (e.g., Achilles tendon [10], flexor tendon [36], rotator cuff [35], patellar [37]). Other studies seeded ADMSCs on decellularized tendon scaffolds, and observed increased tendon marker gene expression, enhanced type-I collagen synthesis, and improved mechanical and biological characteristics [38–40]. However, no previous study has explored the seeding of ADMSCs on SIS for tendon repair.

ADMSCs from subcutaneous adipose tissues are similar to BMSCs in morphology and cell surface markers. Additionally, both cells possess the ability of self-renewal and multilineage potential [33, 41]. Behfar and Nixon et al. [20, 42] managed flexor tendon injury by injection of ADMSCs, and observed improved collagen fiber organization, tendon structure, and yield loads. Park et al. [43] reported the expression of scleraxis and Tenomodulin in cultured ADMSCs treated with IGF-1 and TGFβ or GDF-5. Kryger et al. [44] reported that, ADMSCs and BMSCs behaved similarly in adhesion and proliferation on scaffolds. These suggest ADMSCs to be a possible and less invasively available substitute to BMSCs in tendon tissue engineering.

Compared with the new tendon-like tissue regenerated in Group C, those in Group D and E possessed higher collagen fiber alignment and ultimate loads, suggesting that ADMSCs may have contributed to the production of the new tendon-like tissues. Wang et al. [45] reported that, the BMSCs seeded in SIS significantly increased pancreatic islet function in rats. Hodde et al. [46] found that SIS retained significant bioactivity and had low immunogenicity to support murine fibroblasts attachment and stimulate pheochromocytoma cell differentiation. In a porcine in vivo epicardial study, Chang et al. [8] observed that seeding of BMSCs on SIS reduced the in vivo adaptive immune response to SIS. In the present study, no macrophages were not observed in HE-stained sections surrounding the implanted scaffolds with or without ADMSCs. Additionally, no sign of fibrous encapsulation or scar formation was observed in gross or histological examination. These suggested the absence of evident immune rejection of the scaffolds, which may be explained by several factors. First, decellularized biomaterials were reported to elicit a relatively weak immune rejection [47]. Second, the SIS scaffolds were isolated from young pigs, and biomaterials harvested from young animals are associated with more tissue remodeling response than immune rejection [48]. Moreover, earlier studies have been reported that ADMSCs are relatively immunogenic [49, 50].

Previous studies have reported that, hypoxic pre-conditioning of ADMSCs enhanced their proliferation, stemness, and angiogenic potential [37, 51, 52]. The present study found that, hypoxic pre-conditioning improved histological characteristics, ultimate tensile load, and expression of tendon-related gene markers in the regenerated tendon-like tissue (Groups E vs. D), consistent with an earlier report [53] that hypoxia enhanced the tenocytic differentiation of AMDSCs. The present study did not investigate the molecular mechanisms underlying these improvements, but several possibilities may be suggested. First, the oxygen partial pressure in the adipose tissue is ~ 2–8% [54], and the oxygen tension in the tendon defect area is also low [55]. Thus, pre-conditioning at 2% O_2_ tension may have increased the in vivo viability of ADMSC after grafting into the defect. Second, hypoxic pre-conditioning may have enhanced the potential of ADMSCs to differentiate into tenocytes [51, 56]. Additionally, hypoxia may have upregulated the secretion of cytokines (e.g., TGFs, angiogenic factor, VEGF, SDF-1/CXCR4, FGF), which play roles in tendon regeneration [29].

CM-DiI has been used to label the cell membrane for fluorescence microscopy due to several advantages, and it does not affect the proliferation and differentiation of the cells [57]. The CM-DiI labels can be retained stably on the cell membrane and detected in daughter cells during their proliferation. Moreover, the efficiency of CM-DiI labeling is relatively high. Thus, this procedure has been considered as a convenient and effective method for steadily tracking cells during in vivo experiments [58]. Four weeks after operation, CM-DiI-labeled ADMSCs were detected in the regenerated tissues in Groups D and E, indicating that they were still viable and their cellular morphology could be identified after staining by DAPI. Triple immunofluorescent staining of CM-DiI, DAPI, and MKX further demonstrated that CM-DiI-labeled ADMSCs in both Groups D and E were positive for MKX, a tenocyte-specific protein. This demonstrates that the ADMSCs preconditioned under either hypoxic or normoxic conditions differentiated into tenocyte-like cells. Furthermore, the proportion of the differentiated ADMSCs was higher in the ADMSCs treated by hypoxic preconditioning compared with those receiving normoxic one.

This study involves several limitations. First, the signaling pathways underlying the positive effects of the hypoxic pre-conditioning were not investigated. Second, only single O_2_ pressure, time, and cell seeding dose were used during pre-conditioning; as a result, optimal conditions and curative effects were not established. Moreover, as in vivo study was completed at Week 4, it remains unknown how the regenerated tissue compares with the intact one on a longer time scale. These need to be answered by systematic studies in the future.

## Conclusion

The engineered tendon graft created by seeding ADMSCs on SIS is superior to cell-free SIS.

The hypoxic precondition further improved the expression of tendon-related genes by the seeded ADMSCs in SIS, and increased the rupture load after grafting in the Achilles tendon defects. Finally, ADMSCs seeded on SIS can survive and differentiate into tenocyte-like cells in vivo, and hypoxia may promote the progress of differentiation. These new findings support the application of ADMSCs and hypoxic preconditioning in tendon tissue engineering.

## Data Availability

All data generated or analysed during this study are included in this article.
